# Twelve‐lead electrocardiography identifies the therapeutic target of paroxysmal supraventricular tachycardia

**DOI:** 10.1002/joa3.12261

**Published:** 2019-11-20

**Authors:** Keiji Matsunaga, Tomoko Inoue, Yuichi Miyake, Makoto Ishizawa, Takahisa Noma, Tetsuo Minamino

**Affiliations:** ^1^ Department of Cardiorenal and Cerebrovascular Medicine Faculty of Medicine Kagawa University Kita‐gun Kagawa Japan

**Keywords:** arrhythmia, atrioventricular nodal reentrant tachycardia, atrioventricular reentrant tachycardia, electrocardiography, paroxysmal supraventricular tachycardia

## Abstract

Twelve‐lead electrocardiography provides us the clue about the differential diagnosis between atrioventricular nodal reentrant tachycardia and atrioventricular reentrant tachycardia.

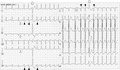

## 1 CASE

**Figure 1:**
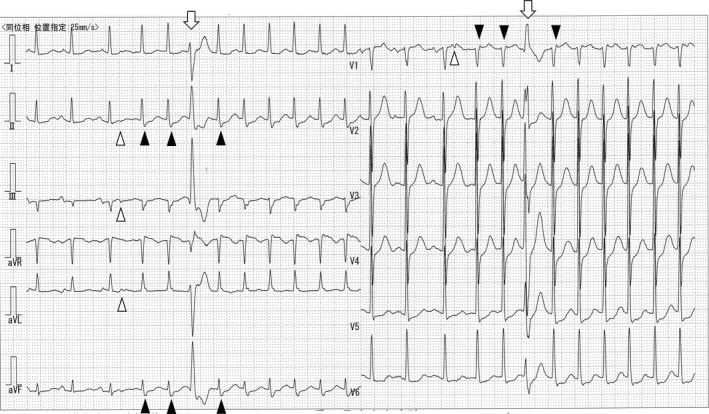
Marked twelve‐lead electrocardiogram at the onset of paroxysmal supraventricular tachycardia (limb leads and precordial leads were in the same phase). White triangles: premature atrial contraction with apparent PQ time prolongation. Black triangles: retrograde P wave very close to the end of the QRS wave. White arrows: the cycle length of this paroxysmal supraventricular tachycardia was not changed by premature ventricular contraction

An 80‐year‐old woman was referred to our institution for the evaluation of sudden onset palpitations. Twelve‐lead electrocardiography (ECG) showed supraventricular tachycardia with a narrow QRS complex, which resolved following intravenous administration of verapamil. However, it recommenced immediately (Figure 1). We sought to elucidate the most likely mechanism of this paroxysmal supraventricular tachycardia (PSVT).

## 2 COMMENTARY

In patients with PSVT, slow‐fast atrioventricular nodal reentrant tachycardia (slow‐fast AVNRT) with a narrow QRS complex is the most common mechanism followed by atrioventricular reentrant tachycardia (AVRT).^1^ Differential diagnosis before performing radiofrequency catheter ablation (RFCA) is important because the possibility of using the Brockenbrough method during RFCA and the risk of an atrioventricular nodal block are different between these two diseases.

This ECG contained three informative findings. First, this tachycardia was started by the trigger of premature atrial contraction (PAC) with apparent PQ time prolongation (Figure 1, white triangles). Since PAC with apparent PQ time prolongation would mean that this patient would have dual AV nodal pathways, called fast pathway and slow pathway, this finding suggested that the mechanism of this PSVT would be slow‐fast AVNRT.

Second, at the onset of and during PSVT, retrograde P waves were observed on this ECG (Figure 1, black triangles). Since the retrograde P wave was just at the end of the QRS wave and showed “terminal QRS notching in lead V1”,^2^ this finding strongly suggested that the mechanism of this PSVT would be slow‐fast AVNRT.

Third, the cycle length of this PSVT was not changed by premature ventricular contraction (PVC; Figure 1, white arrows). Since AVRT is an arrhythmia that includes the ventricle in a tachycardia circuit, the cycle length of AVRT is sometimes changed by PVC. Therefore, this finding suggested that this PSVT did not include the ventricle in the tachycardia circuit, indicating that the mechanism of this PSVT would be slow‐fast AVNRT.

These characteristic findings strongly suggested the mechanism of PSVT before the electrophysiologic study (EPS) and guided the strategy of RFCA. Thereafter, this patient underwent an EPS and RFCA for the slow pathway, which is the recommended type of ablation for slow‐fast AVNRT, and PSVT has not recurred since.

**Figure 2:**
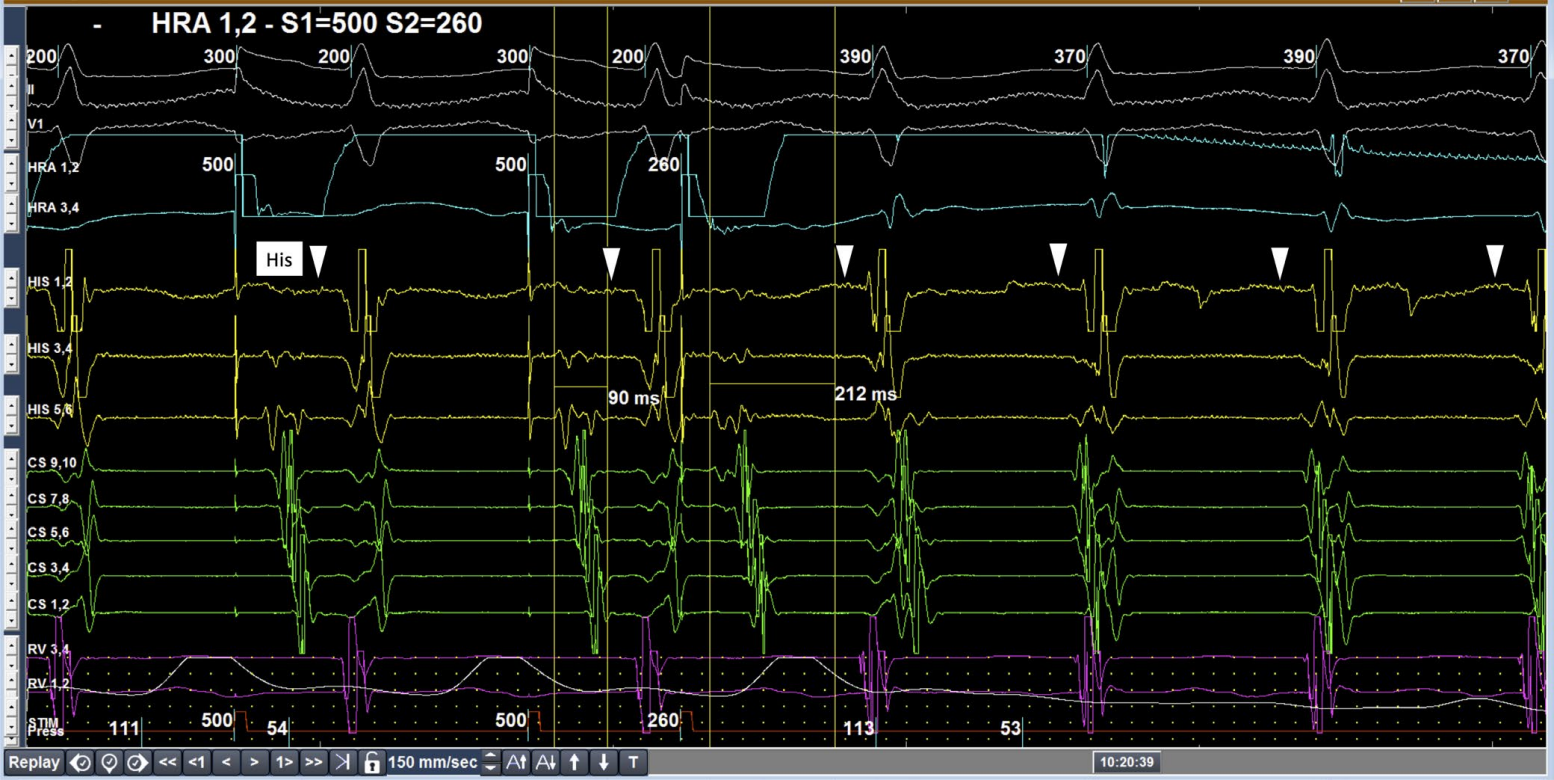
Intracardiac electrocardiogram showing the “jump up phenomenon” and the onset of slow‐fast atrioventricular nodal reentrant tachycardia

In this manuscript, we present the ECG of typical slow‐fast AVNRT and its interpretation. It is possible that some students may face difficulty in the interpretation of this ECG. In such a scenario, EPS is a potent tool that gives us the therapeutic clue to the diagnosis of the PSVT; in other words, EPS clarifies the findings that cannot be confirmed from the electrocardiogram alone. For example, Figure 2 shows the intracardiac electrocardiogram of the “jump up phenomenon” and the onset of slow‐fast AVNRT.

Since the detailed mechanism is quite technical, the jump up phenomenon seen on EPS confirms the presence of dual AV nodal pathways, called fast pathway and slow pathway. Therefore, EPS clarified the findings, which did not become clear purely from an electrocardiogram.

In view of this detailed information deducible from the ECG, we anticipate a greater degree of interest in arrhythmias on the part of students.

## CONFLICT OF INTEREST

The authors declare no conflict of interests for this article.
